# Intramuscular Delivery of Gene Therapy for Targeting the Nervous System

**DOI:** 10.3389/fnmol.2020.00129

**Published:** 2020-07-17

**Authors:** Andrew P. Tosolini, James N. Sleigh

**Affiliations:** ^1^Department of Neuromuscular Diseases, UCL Queen Square Institute of Neurology, University College London, London, United Kingdom; ^2^UK Dementia Research Institute, University College London, London, United Kingdom

**Keywords:** adenovirus (AdV), adeno-associated virus (AAV), axonal transport, lentivirus, motor neuron, neuromuscular junction (NMJ), peripheral nerve, sensory neuron

## Abstract

Virus-mediated gene therapy has the potential to deliver exogenous genetic material into specific cell types to promote survival and counteract disease. This is particularly enticing for neuronal conditions, as the nervous system is renowned for its intransigence to therapeutic targeting. Administration of gene therapy viruses into skeletal muscle, where distal terminals of motor and sensory neurons reside, has been shown to result in extensive transduction of cells within the spinal cord, brainstem, and sensory ganglia. This route is minimally invasive and therefore clinically relevant for gene therapy targeting to peripheral nerve soma. For successful transgene expression, viruses administered into muscle must undergo a series of processes, including host cell interaction and internalization, intracellular sorting, long-range retrograde axonal transport, endosomal liberation, and nuclear import. In this review article, we outline key characteristics of major gene therapy viruses—adenovirus, adeno-associated virus (AAV), and lentivirus—and summarize the mechanisms regulating important steps in the virus journey from binding at peripheral nerve terminals to nuclear delivery. Additionally, we describe how neuropathology can negatively influence these pathways, and conclude by discussing opportunities to optimize the intramuscular administration route to maximize gene delivery and thus therapeutic potential.

## Introduction

With thousands of clinical trials to date, gene therapy is a flourishing strategy with great promise for the treatment of diseases impacting the nervous system. Indeed, virus-mediated gene therapies have now been approved by the FDA in the US for *RPE65*-associated retinal dystrophy (*voretigene neparvovec* marketed as Luxturna) and *SMN1*-linked spinal muscular atrophy (SMA; *onasemnogene abeparvovec* marketed as Zolgenmsa), as well as non-neuronal conditions (High and Roncarolo, 2019). Gene therapy viruses are non-replicating, but still hijack host cell machinery to express transgenes of interest in the nucleus. Crucially, some viral vectors (i.e., viruses specifically used to deliver genetic material into cells) have the potential to circumvent the blood-brain- (BBB) and blood-spinal cord barriers (BSCB) when intravenously injected. Similarly, direct injection of viruses into the cerebrospinal fluid (e.g., *via* lumbar puncture in humans) also permits targeting of the peripheral (PNS) and central nervous systems (CNS). These two administration routes for neuronal delivery have been extensively covered in recent reviews (Hocquemiller et al., 2016; Deverman et al., 2018; Hudry and Vandenberghe, 2019). A complementary, and perhaps sometimes superior (Benkhelifa-Ziyyat et al., 2013), method to introduce genetic material into select neuronal populations is by virus administration into muscle, which is the focus of this review. Muscles contain the synaptic connection between lower motor neurons and muscle fibers, i.e., the neuromuscular junction (NMJ), as well as specialized sensory nerve endings (e.g., muscle spindles). Viruses can be internalized into peripheral nerve terminals and subsequently retrogradely transported along axons to deliver viral payloads into corresponding motor and sensory neurons, with scope for widespread transfer to additional cells throughout the spinal cord and brain (Benkhelifa-Ziyyat et al., 2013; Chen et al., 2020).

The NMJ is a tripartite synapse comprised of a pre-synaptic motor nerve terminal, a post-synaptic muscle fiber, and several terminal Schwann cells (Li et al., 2018a). Moreover, the synaptic cleft consists of a complex and dynamic extracellular matrix (ECM) that contributes to receptor translocation and internalization of a variety of molecules (Heikkinen et al., 2020). Targeting muscles with viruses can transduce all three cellular constituents of the NMJ (Mazarakis et al., 2001; Homs et al., 2011)—by “transduction,” we mean the introduction of genetic material into target cells. Furthermore, uptake at sensory nerve terminals can lead to transgene expression in dorsal root ganglia (DRG), trigeminal ganglia, and dorsal horn nerve fibers (Watson et al., 2016; Chen et al., 2020). When injected into a muscle, viruses are close to nerve endings for longer periods and at higher concentrations than when systemically injected. Moreover, limiting widespread virus distribution is likely to decrease safety risks due to immunogenicity or toxicity, while possible negative effects caused by central injections will be avoided. Hence, targeting muscle may prove to be a useful method to introduce viral vectors to certain central and peripheral neurons and/or glia.

For this strategy to be exploited, viruses must undergo several major processes, including host cell binding, internalization, intracellular sorting, and retrograde axonal trafficking to neuronal soma before nuclear entry. In this review article, we outline these mechanisms for major gene therapy viruses—adenovirus (AdV), adeno-associated virus (AAV) and lentivirus (LV; Table 1)—with a focus on peripheral neurons. We also comment on the impact of neuropathology on using intramuscular virus injection as an administration route. To conclude, we discuss opportunities to optimize gene therapy delivery to muscle for nervous system targeting.

**Table 1:** Gene therapy virus characteristics.

	Adenovirus	AAV	Lentivirus
Size (nm)	≈90	≈25	80–120
Genome type	dsDNA	ssDNA	ssRNA
Packaging capacity (kb)	≈8^*^	≈4.7^#^	≈8
Enveloped	No	No	Yes
Integration	No	No	Yes
Expression	Transient	Persistent	Persistent
Immunogenicity	High	Moderate	Low

Adapted from Worgall and Crystal (2014); Lee et al. (2017); Kariyawasam et al. (2020). ^*^This is increased to ≈36 kb in the “helper-dependent” human AdV serotype 5. ^#^This is halved in self-complementary AAV. *ds*, double-stranded; *ss*, single-stranded.

## Gene Therapy Viruses

### Adenovirus

First isolated in the 1950s, AdVs are non-enveloped, double-stranded DNA viruses with an icosahedral-shaped capsid comprised mainly of hexon and penton capsomeres (Greber and Flatt, 2019). *Adenoviridae* encompasses more than 300 different vertebrate-infecting types, including seven human AdV (HAdV) species (A to G) currently comprised of ≈80 types classified by serology or sequencing. HAdVs primarily cause ocular, gastrointestinal, or respiratory infections (Ghebremedhin, 2014). It is estimated that more than 80% of the human population has been exposed to HAdV and develop type-specific humoral and cross-reactive cellular immunity (Ahi et al., 2011), hence, for utilization as a gene therapy vector, strategies to circumvent the host immune response have been examined (Duffy et al., 2012). In the 1990s, AdV became the first gene therapy virus to be tested in human clinical trials and currently remains the most investigated (Lee et al., 2017). The more common human serotypes 2 and 5 belonging to species C have been the focus for gene therapy development. E1/E3-deleted AdVs have a relatively large packaging capacity of ≈8 kb, can transduce many different cell types, and form episomes rather than integrating into the host genome. Moreover, AdVs can be efficiently produced in large, concentrated quantities. In some hosts and some organs, transgene expression using AdV can be transient, likely due to host-specific responses, while in other cases, transgene expression remains robust for months (Li et al., 2016). In this regard, the transient expression can be advantageous for scenarios requiring short-term upregulation of therapeutic genes and for limiting deleterious consequences that may arise from long-term expression (discussed in Tosolini and Morris, 2016b). However, the transgene capacity of AdV can be increased up to ≈36 kb by removing essential elements and exogenously providing them for *in vitro* packaging, and with this approach, they lack the elements that usually activate host immunity, which can thereby facilitate prolonged-expression (Ricobaraza et al., 2020). Permitting much broader options for transgene incorporation, this expansive packaging capacity is one major advantage of AdV over other viral vectors.

AdVs display broad cell and tissue tropisms mediated by the interaction between their capsid and specific cellular receptors (Arnberg, 2012). Capsid modification, for instance by altering the virus genome or adding ligands, can widen or narrow tissue specificity depending on the required strategy (Worgall and Crystal, 2014). Direct intracranial injection of HAdV has been shown to result in the transduction of several different neuronal and non-neuronal cell types in the rodent CNS (Akli et al., 1993; Davidson et al., 1993; Le Gal La Salle et al., 1993). Furthermore, intramuscular administration of AdVs can result in their uptake at rodent NMJs and sensory terminals before retrograde transport to cell bodies (Finiels et al., 1995; Ghadge et al., 1995; Tosolini and Morris, 2016a), which is a viable strategy to counteract neuromuscular disease (Haase et al., 1998; Acsadi et al., 2002) and peripheral nerve injury (Giménez y Ribotta et al., 1997; Baumgartner and Shine, 1998). Of note, the canine adenovirus serotype 2 (CAV-2; also known as CAdV-2), which can cause mild respiratory infections in *Canidae*, has become the AdV of choice for neuronal transduction (Del Rio et al., 2019). Due to possessing greater specificity in host cell receptor binding than HAdVs, CAV-2 preferentially targets neurons (Soudais et al., 2001). Furthermore, it is efficiently retrogradely transported along axons (Salinas et al., 2009), while a helper-dependent CAV-2 has been shown to drive transgene expression in the rodent CNS for over a year (Soudais et al., 2004). CAV-2 injection into craniofacial muscles of rhesus monkeys caused robust motor neuron transduction (Bohlen et al., 2019), while intramuscular administration in rats results in superior motor neuron uptake and transport compared to AdV serotype 5 (Soudais et al., 2001), which together highlight the potential of CAV-2 for motor neuron targeting *via* skeletal muscle.

### Adeno-Associated Virus

Belonging to the *Dependoparvovirus* genus and thus needing factors from helper viruses (e.g., AdV) to replicate, AAVs are non-enveloped, single-stranded DNA viruses discovered as AdV preparation contaminants (Zinn and Vandenberghe, 2014). More than 100 natural AAV variants, including 13 serotypes from primates, have been identified, each with differing tissue tropisms, transduction efficiencies, and antigenicities, all resulting from their distinct protein capsids (Zincarelli et al., 2008; Srivastava, 2016). Additional synthetic AAV subtypes have been derived/engineered in the laboratory to optimize these features for gene transfer (Kotterman and Schaffer, 2014). Impinging considerably upon its tractability, the packaging capacity of AAV is limited to ≈4.7 kb, which is halved in the more rapidly expressing self-complementary AAV (for simplicity, we refer to single-stranded and self-complementary AAV as one), although DNA delivery across separate AAV particles is possible (Patel et al., 2019). In most cases, AAV vectors induce limited immunogenicity in naïve hosts (Ronzitti et al., 2020), and have a good safety record, although there may be toxicity issues when administered at high doses (Hinderer et al., 2018). However, the AAV vector effect on brain homeostasis has not been completely addressed and is an important consideration (He et al., 2019). Forming stable, non-replicating episomes for sustained transgene expression, AAV is largely non-integrating (Schnepp et al., 2005), although insertional mutagenesis has been reported (Chandler et al., 2017). These combined features have led to AAV becoming the premier clinical gene therapy vector and its recent regulatory approval for the treatment of several conditions (High and Roncarolo, 2019). However, AAV gene therapy is not entirely infallible, as wild type AAV infections have been linked with human disease (Nault et al., 2016); however, potential solutions to overcome these and other concerns to drive human AAV gene therapy are continuing (Colella et al., 2018). Nonetheless, many more clinical trials of AAV-mediated gene therapy are ongoing or planned, including several involving intramuscular administration (although not necessarily for neuronal transduction).

AAVs have been used for many years in the laboratory to drive transgene expression in the nervous system (Hudry and Vandenberghe, 2019). Due to its ability to cross the BBB, AAV serotype 9 (AAV9) has become the principal serotype for CNS-targeting upon systemic administration (Foust et al., 2009; Bevan et al., 2011; Samaranch et al., 2012), although superior serotypes, such as AAVrh10, have also emerged (Tanguy et al., 2015). However, cell binding and transduction can change with age (Chakrabarty et al., 2013), thus engineered serotypes with greater neuronal tropism, at least in mice, are being developed (Choudhury et al., 2016; Deverman et al., 2016). Nervous system delivery has also been achieved by AAV injection into muscle; intramuscular administration of several AAV serotypes (e.g., AAV2, AAV9) results in AAV uptake into the motor and sensory neurons in rodents (Hollis Ii et al., 2008; Zheng et al., 2010; Benkhelifa-Ziyyat et al., 2013; Jan et al., 2019; Chen et al., 2020) and motor neurons in non-human primates (Towne et al., 2010). Consequently, this method of gene delivery has proven beneficial in mouse models of motor neuron diseases amyotrophic lateral sclerosis (ALS), and SMA (Tosolini and Sleigh, 2017). Increasing the possible clinical applicability of AAV, single intramuscular injections of rAAV2-retro, a newly evolved variant with robust retrograde transport capacity (Tervo et al., 2016), were recently shown to result in broad transgene expression across ipsilateral and contralateral motor neurons along the length of the spinal cord, as well as brainstem motor nuclei, DRG, trigeminal ganglia and dorsal horn nerve fibers (Chen et al., 2020). Importantly, AAV targeting of peripheral neurons is therefore not limited to those cells innervating the injected muscle.

### Lentivirus

Belonging to the *Retroviridae* family, LV possesses a single-stranded RNA genome and can infect both dividing and non-dividing cells (Parr-Brownlie et al., 2015). LV is an enveloped virus with a packaging capacity of ≈8 kb and it relies on reverse transcription of its single-stranded RNA genome to generate corresponding double-stranded DNA for integration into the host genome (Mátrai et al., 2010). This provides benefits of long-term transgene expression and inheritance of genetic material in dividing cells; however, integration also has the major disadvantage that it can disrupt host gene function through insertional mutagenesis, which poses a safety risk. Incorporation into the host genome is not random, as there are preferential sites and conditions for integration (e.g., highly expressed and intron-rich genes), but it is unpredictable (Lesbats et al., 2016). Nonetheless, this has not prevented several LV-mediated gene therapies being approved for human use, albeit being utilized for *ex vivo* modification of autologous immune cells (High and Roncarolo, 2019). For gene delivery, essential viral coding regions (e.g., *gag*, *pol*, and *env*) are removed from the LV genome, and instead provided by separate expression plasmids for *in vitro* packaging (Milone and O’Doherty, 2018). This removal of viral genes ensures that the immunogenicity of LV is relatively low, although not absent (Annoni et al., 2019).

LVs are typically derived from primate or non-primate immunodeficiency viruses [e.g., human immunodeficiency virus type 1 (HIV-1) or equine infectious anemia virus (EIAV)]. LV tropism is mediated by the viral envelope, which is engineered to include glycoproteins from other enveloped viruses in a process called pseudotyping (Cronin et al., 2005). The most common virus used to pseudotype LV is the vesicular stomatitis virus (VSV), but heterologous envelope proteins from many other viruses have been used to target LV to particular cells and tissues, e.g., measles virus, murine leukemia virus and influenza viruses (Joglekar and Sandoval, 2017). The VSV glycoprotein (VSV-G) binds to a widely expressed receptor, leading to broad tropism when integrated into the LV envelope. In contrast, LVs pseudotyped with rabies virus (RV) display greater neuronal selectivity and have been shown to aid efficient transduction of neurons both *in vitro* and *in vivo*. Compared to VSV, LV pseudotyped with RV glycoprotein (LV-RV) shows superior neuronal transduction and transport when injected into the rat striatum and spinal cord (Mazarakis et al., 2001). A similar high efficiency has been reported when injected into the primate brain (Kato et al., 2007), while distal uptake and efficient retrograde trafficking occurs in rodent primary motor neurons (Hislop et al., 2014). Moreover, LV-RV administration into gastrocnemius muscle results in effective transgene expression in spinal cord motor neurons, while LV-VSV remains restricted to the muscle injection site (Mazarakis et al., 2001), which was confirmed with additional RV strains (Wong et al., 2004; Mentis et al., 2006). Pseudotyping with several different hybrid glycoproteins has since shown improved targeting of motor neurons when delivered to muscle, which can be further enhanced by the coupling of antibodies against NMJ receptors to the virus surface (Hirano et al., 2013; Eleftheriadou et al., 2014). As a consequence, numerous different LV-mediated therapeutic strategies that target motor neurons *via* muscle have proven successful in mouse models of ALS and SMA (Azzouz et al., 2004a,b; Ralph et al., 2005; Raoul et al., 2005; Benkler et al., 2016; Eleftheriadou et al., 2016).

## From Virus Binding to Nuclear Entry

**Figure 1:**
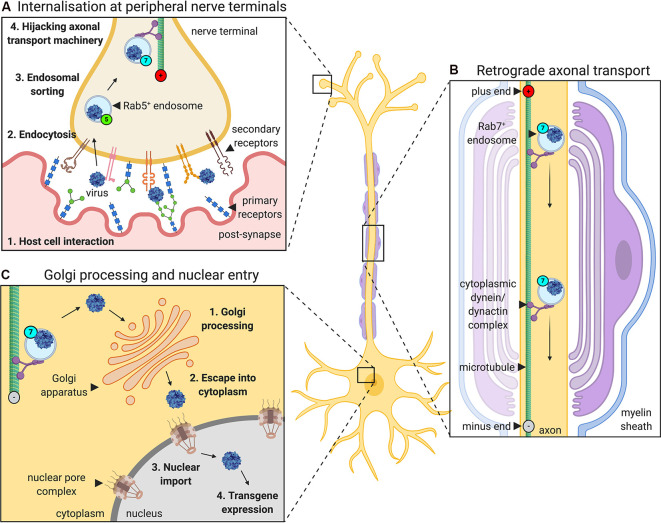
The journey of gene therapy viruses from peripheral nerve terminals to the nucleus. Viruses used to deliver gene therapy must access cell nuclei to express their packaged genetic material. When administered into muscles for targeting of peripheral nerve somas, viruses such as adenovirus, adeno-associated virus (AAV) and lentivirus, undergo a series of processes that aid their transfer from the periphery to CNS (depicted here using AAV as an example). **(A)** First, the virus interacts with specific host cell surfaces. This entails primary receptor binding (e.g., glycans) followed by internalization, which is often mediated, at least in part, by a secondary receptor (e.g., AAV receptor, AAVR or fibroblast growth factor receptor, FGFR). Internalization at nerve terminals is regulated by a variety of endocytic pathways. Post-internalisation, viruses hijack the Rab GTPase-mediated endosomal sorting system, transitioning through Rab5-positive early endosomes to non-acidic Rab7-positive late endosomes. **(B)** Virus-containing Rab7-positive signaling endosomes are actively transported along microtubules by cytoplasmic dynein-dynactin complexes towards nerve cell bodies (i.e., retrogradely). **(C)** At the neuronal soma, viruses escape endosomes and are processed, sometimes through the Golgi apparatus, before entry into the nucleus (e.g., *via* the nuclear pore complex), where the virus can begin to drive transgene expression.

For viruses injected into a muscle to express transgenes in neurons, they must undergo a series of events: host cell binding and internalization, intracellular sorting, retrograde axonal transport, liberation from the transporting structure/organelle and nuclear entry (Figure 1). AdV, AAV, and LV rely on the same or similar mechanisms for several parts of this journey which are also shared by botulinum and tetanus neurotoxins (Surana et al., 2018). For instance, they all hijack retrograde axonal transport (Merino-Gracia et al., 2011), which is dependent on active, processive movement along microtubules by the motor protein complex cytoplasmic dynein-dynactin (Schiavo et al., 2013). By trafficking towards the stable minus ends of the microtubule, which are located at the cell body end of an axon, cytoplasmic dynein enables long-range retrograde delivery of cargoes, such as autophagosomes and neurotrophin-containing signaling endosomes. Additionally, the Rab (Ras-related proteins in the brain) GTPase protein family is specifically required for signaling endosome trafficking (Villarroel-Campos et al., 2018). Target tissue-derived (e.g., muscle) neurotrophins transition from early Rab5-positive endosomes into retrogradely transported Rab7-positive signaling endosomes (Deinhardt et al., 2006). Unlike in the canonical endolysosomal pathway, retrograde Rab7-endosomes within axons display a tightly regulated neutral pH value that is maintained during transport (Bohnert and Schiavo, 2005). All three gene therapy viruses have been shown to localize to these axonal Rab7-endosomes, indicating that they share a common compartment when voyaging to the nucleus. Retrograde trafficking is a rapid and constitutive process that delivers large quantities of endosomes to the motor and sensory soma; it is thus unlikely to be a rate-limiting step in virus transgene expression. Rather, idiosyncratic aspects of the journey of each virus, e.g., binding to specific receptors or endosomal liberation at the cell body, probably have a greater impact on overall transduction efficiency.

Highlighting similarities and differences, we now describe the individual journeys that each virus must take to migrate from muscle to peripheral nerve soma for transgene expression.

### Adenovirus

Similar to most viruses, AdV is typically internalized in a two-step, receptor-mediated fashion that is dependent on the viral capsid, although non-specific, large-scale internalization has also been reported (Meier et al., 2002). Primary receptors that mediate AdV attachment to cells include, heparan sulfate proteoglycans, CD46, and sialic acid, which selectively interact with different serotypes (Arnberg, 2012); however, the appears to be the major initial binding partner for AdVs (Bergelson et al., 1997; Arnberg, 2012). Coxsackie and adenovirus receptor (CAR) is a widely expressed cell adhesion protein critical for heart development (Dorner et al., 2005), and is involved in neurogenesis through its synaptic expression throughout the mature brain (Zussy et al., 2016). CAR serves as the primary receptor for several different HAdV species (i.e., A, C-F) and serotypes, including 2 and 5, as well as CAV-2 (Arnberg, 2012; Loustalot et al., 2016). The second step of AdV internalization (i.e., entry) is facilitated by penton capsomere binding to members of the integrin receptor family, e.g., α_V_β_3_ and α_V_β_5_ (Wickham et al., 1993). Facilitating cell-to-cell and cell-to-ECM interactions, integrins are expressed in a tissue-specific fashion and can in some instances mediate AdV attachment in the absence of CAR (Huang et al., 1996).

Despite extensive knowledge on AdV receptors, relatively little is known about the specific entry of AdV at the NMJ or sensory nerve terminals. Intramuscular injections of AdV result in the targeting of both muscle fibers and innervating motor neurons in juvenile and adult mice (Tosolini and Morris, 2016a), which is consistent with the reported expression of CAR in muscle fibers (Nalbantoglu et al., 1999) and at both mouse and human NMJs (Shaw et al., 2004; Sinnreich et al., 2005). However, one of the major issues with AdV-mediated gene therapy is the relatively poor transduction of neurons in adults compared to young mice, including upon intramuscular injection (Acsadi et al., 1994; Huard et al., 1995; Tosolini and Morris, 2016a). This is somewhat unsurprising as CAR is downregulated post-natally in several neuronal subtypes (Hotta et al., 2003) and muscle (Nalbantoglu et al., 1999). Indeed, CAR is highly expressed in immature skeletal muscle fibers but is drastically downregulated after birth (Nalbantoglu et al., 1999) becoming restricted to the NMJ (Shaw et al., 2004; Sinnreich et al., 2005). Nevertheless, to better understand the limited uptake of AdV into adult motor neurons, further investigation is required to provide a thorough longitudinal assessment of CAR levels at post-natal neuromuscular synapses. Upon muscle damage caused by Duchenne muscular dystrophy or polymyositis, CAR expression increases within muscle fibers and co-localizes with markers of regeneration (Sinnreich et al., 2005); given the parallels between mechanisms of muscle development and regeneration, this suggests that CAR may indeed be developmentally regulated at the NMJ and serve in the synaptic response to regeneration (Sinnreich et al., 2005).

After binding to CAR, AdVs are internalized and processed in a cell type-dependent manner. Experiments in immortalized non-neuronal cells describe AdV internalization into endosomes *via* clathrin-coated pits (Meier et al., 2002) and subsequent endosomal liberation *via* acidification (Leopold et al., 1998). The intracellular domain of CAR plays a critical role in this by recruiting the endocytic machinery and influencing subsequent intracellular AdV trafficking (Loustalot et al., 2015). AdVs are then transported towards the nucleus by cytoplasmic dynein-mediated trafficking along with the microtubule network (Kelkar et al., 2004), impairments in which drastically disrupt this nuclear targeting (Suomalainen et al., 1999; Leopold et al., 2000). The AdV capsid directly interacts with cytoplasmic dynein *via* hexon capsomeres (Bremner et al., 2009), suggesting that in non-neuronal cells AdVs are transported as “naked particles” rather than in membrane-bound organelles (e.g., endosomes; Scherer et al., 2020). Moreover, this interaction appears to be dependent on exposure to low pH, suggesting that AdV binding to the motor protein is primed by transition through the early endosomal system (Bremner et al., 2009). AdV serotype 5 has also been shown to interact with the Kif5B subunit of kinesin-1, a motor protein that drives transport in the opposite direction to cytoplasmic dynein (i.e., towards dynamic plus ends), possibly as an evolutionary strategy for increased cellular exploration (Zhou et al., 2018).

In primary neurons, AdVs are also internalized in a CAR-dependent manner (Loustalot et al., 2016), facilitated by CAR enrichment in actin-domains of neuronal growth cones as well as lipid rafts (Huang et al., 2007). Internalization occurs through a lipid microdomain-, actin- and dynamin-dependent manner before the receptors are eventually targeted for lysosomal degradation (Salinas et al., 2014). The major difference between neuronal and non-neuronal AdV trafficking is that in neurons, CAR does not undergo lysis during intracellular sorting, and is instead transported to the neuronal soma as part of non-acidic, Rab7-positive endosomes, thus preventing pH-induced conformational changes to the AdV capsid and restricting endosomal liberation (Salinas et al., 2009). CAR-positive organelles favor the retrograde direction but can also be anterogradely transported by kinesin motor proteins (Salinas et al., 2009). Again confirming the essential nature of transport to AdV migration, *in vivo* pharmacological blockade of microtubule dynamics inhibits the delivery of AdV to the neuron (Boulis et al., 2003). Once in the soma, AdV accesses the nucleus at the nuclear pore complex *via* histone H1 (Trotman et al., 2001) or the nucleoporin receptors (Trotman et al., 2001; Cassany et al., 2015), with the route also appearing to be cell type-dependent (Kremer and Nemerow, 2015).

### Adeno-Associated Virus

AAV also gains cellular access *via* a two-step process involving primary cell surface receptors with a secondary receptor mediating entry. Negatively charged glycans or glycoconjugates serve as primary attractants with which AAVs initially interact allowing extracellular viral accumulation and co-receptor access. These include heparan sulfate proteoglycans for AAV2, AAV3, AAV6 and AAV13, N-terminal galactose for AAV9, and specific N- and O-linked sialic acid moieties for AAV1, AAV4, AAV5 and AAV6 (Huang et al., 2014). The wide expression of surface glycans, including in neuronal extracellular matrices (Broadie et al., 2011; Singhal and Martin, 2011), explains the broad infectivity of AAV, while glycan diversity and relative density likely dictates selectivity of AAV serotype tropism.

Several serotype-specific co-receptors have also been identified that after glycan binding, facilitate AAV uptake. These co-receptors include fibroblast growth factor receptor (FGFR) and hepatocyte growth factor receptor (HGFR) for both AAV2 and AAV3, platelet-derived growth factor receptor (PDGF) for AAV5, and epidermal growth factor receptor (EGFR) for AAV6 (Madigan and Asokan, 2016). Signaling through each of these receptors has been linked to NMJ formation/function (Zhao et al., 1999; Li et al., 2012; Taetzsch et al., 2018), consistent with their synaptic availability. Additional receptors have been identified for engineered serotypes contributing to distinct tropisms (Hordeaux et al., 2019; Huang et al., 2019). However, a common receptor required for endocytosis of most natural primate AAV serotypes was recently identified (Pillay et al., 2016). Originally called KIAA0319L and linked with dyslexia and functions of neuronal migration and axon guidance (Poon et al., 2011), the AAV receptor (AAVR) possesses an N-terminal MANSC domain, several immunoglobulin-like PKD domains, a C6 domain, and a transmembrane region before a short C-terminal tail (Poon et al., 2011). As expected given the broad cellular and tissue infectivity of AAV, AAVR is expressed across many human tissues, including muscle and nerve, and can be found as several spliced variants and post-translationally modified isoforms (Poon et al., 2011; Gostic et al., 2019). AAVR knockout rendered mammalian HeLa cells highly resistant to infection with AAV serotypes 1, 2, 3b, 5, 6, 8, and 9, with a similar finding in AAV9-injected AAVR knockout mice *in vivo* (Pillay et al., 2016). The removal of AAVR resulted in no obvious phenotype, suggesting that AAVR is non-essential or there is genetic compensation. In subsequent work from the same group and others, AAV serotypes have been shown to differentially interact with AAVR PKD domains (Pillay et al., 2017; Zhang et al., 2019), while AAV4 gains full cellular access in absence of the receptor, suggesting that some serotypes can utilize non-AAVR internalization pathways (Dudek et al., 2018). In immortalized cells, AAVR localizes to the cytoplasm and perinuclear region where it associates with the Golgi network (Poon et al., 2011; Pillay et al., 2016). Several hypotheses as to where exactly AAV interacts with AAVR have been put forward, including on the cell surface, in the endolysosomal system and at the Golgi apparatus; however, this requires further clarification (Summerford et al., 2016; Pillay and Carette, 2017).

Data are supporting several distinct AAV internalization mechanisms, including clathrin-dependent endocytosis (Uhrig et al., 2012), caveolar endocytosis (Sanlioglu et al., 2000), and the clathrin-independent carriers and GPI-enriched endocytic compartments (CLIC/GEEC) pathway (Nonnenmacher and Weber, 2011). However, not all routes result in an efficient delivery to the nucleus, rather they traffic AAV through unproductive paths leading to a viral cul-de-sac (Nonnenmacher and Weber, 2012; Pillay and Carette, 2017); only ≈30% of internalized AAV is estimated to enter the nucleus (Zhong et al., 2008; Xiao et al., 2012). Nonetheless, there are distinctions in AAV uptake depending on cell type and serotype (Weinberg et al., 2014), thus future work identifying neuronal-specific internalization mechanisms is required.

Upon cellular entry, AAVs have been reported to be retrogradely transported from the cell surface to Golgi in a syntaxin 5-dependent mechanism (Nonnenmacher et al., 2015), before escaping into the cytoplasm and entering into the nucleus *via* the nuclear pore complex (Nicolson and Samulski, 2014). However, before reaching the Golgi, AAV must transit through various acidic endosomal compartments to drive pH- and cathepsin-mediated conformational changes in the capsid (Akache et al., 2007; Salganik et al., 2012). Indeed, the passage of AAV through the endosome to Golgi system appears to be necessary for transgene expression, as AAV directly injected into cytosol do not migrate to the nucleus (Sonntag et al., 2006). AAV has been reported to localize to Rab5-, Rab7-, and Rab11-positive (recycling) endosomes (Berry and Asokan, 2016), and, as expected, requires a functioning microtubule network for transport (Xiao and Samulski, 2012). Nevertheless, its exact route through the cell requires further elucidation, especially its transit through long and highly polarized peripheral nerves, as little data have been generated in neurons.

That being said, there is ample indirect evidence that AAVs are transported in axons *in vivo* both in peripheral (Hollis Ii et al., 2008; Towne et al., 2010; Zheng et al., 2010; Benkhelifa-Ziyyat et al., 2013; Jan et al., 2019) and CNS (Salegio et al., 2013; Castle et al., 2014a,b) neurons, suggesting the availability of AAV receptors and uptake mechanisms; however, observations of AAV being actively trafficked are limited. Nevertheless, peripherally administered AAV likely hijacks Rab-positive endosomes in peripheral nerves to reach the CNS, like that of AdV. Indeed, in primary cortical neurons grown in microfluidic chambers to separate axons and soma, AAV9 was shown to localize in a time-dependent fashion to several different endosomes/vesicles (e.g., Rab5-, Rab7-, Rab11-positive; Castle et al., 2014b). AAV9 internalized at axon tips was retrogradely transported in cytoplasmic dynein-dynactin-driven Rab7-positive endosomes and was subsequently capable of inducing transgene expression post-transition through the Golgi (Castle et al., 2014b). Moreover, in a companion study it was shown that AAV1, AAV8, and AAV9 share the same intra-axonal compartment when being transported in primary cortical neurons, indicating that once they have gained access to the endosomal sorting system, AAV serotypes harness common axonal transport mechanisms (Castle et al., 2014a). However, direct evidence from the motor and sensory neurons remains unavailable.

### Lentivirus

LV tropism is dictated by the envelope glycoproteins with which it has been pseudotyped (Cronin et al., 2005). VSV-G interacts with the low-density lipoprotein receptor (LDLR; Finkelshtein et al., 2013). LDLR mediates uptake of cholesterol-rich LDL and is broadly expressed, thus LV-VSV is pan-tropic. A measure of cell-type selectivity can be achieved with cell/tissue-specific promoters, which is a strategy used with all three gene therapy viruses. For example, LV-VSV combined with an *hGFAP* promoter induces astrocytic expression, whereas LV-VSV with an *rNSE* promoter selectively expresses in neurons (Jakobsson et al., 2003). Alternatively, envelope modification coupled with surface antibody-mediated targeting can confer tissue specificity and improve virus uptake (Yang et al., 2006; Eleftheriadou et al., 2014). In contrast, LV-RV interacts with receptors that are predominantly expressed by neurons, including the pan-neurotrophin receptor p75^NTR^ (Tuffereau et al., 1998), neuronal cell adhesion molecule (NCAM; Thoulouze et al., 1998) and nicotinic acetylcholine receptor (nAChR; Hanham et al., 1993). p75^NTR^ non-selectively binds to all neurotrophins (i.e., BDNF, NGF, NT-3, and NT-4/5) and, depending on the active co-receptor, can activate both pro-survival or pro-death signaling (Gentry et al., 2004). NCAM is an immunoglobulin-like glycoprotein that mediates cell-to-cell contact and functions in adhesion, guidance, and differentiation during neuronal growth (Weledji and Assob, 2014). nAChRs bind to the excitatory neurotransmitter acetylcholine secreted into the synaptic cleft to facilitate depolarization of the postsynaptic cell. All three LV-RV receptors are integral constituents of the NMJ (although nAChRs are post-synaptic), explaining the efficient *in vivo* uptake into motor neurons of these RV pseudotyped viruses when injected into a muscle (Mazarakis et al., 2001; Azzouz et al., 2004a,b; Wong et al., 2004).

After receptor-mediated internalization, most likely in clathrin-coated pits as dictated by their neuronal receptors (i.e., p75^NTR^; Bronfman et al., 2003), RV-LVs migrate through the endolysosomal system transitioning from Rab5-positive early endosomes to the non-acidic Rab7-positive compartment (Hislop et al., 2014). In non-neuronal cells, endosome acidification causes a conformational change in LV glycoproteins, which initiates membrane fusion between the viral envelope and endosome membrane to permit the escape of the virus into the cytoplasm (Gaudin et al., 1993; Gaudin, 2000). However, in neurons, LVs are retrogradely transported within neutral Rab7-positive signaling endosomes towards peripheral nerve cell bodies through the same motor protein-driven process as AdV and AAV. In rat primary motor neuron cultures, LV-RV was shown to co-localize in axons with all three receptors (i.e., p75^NTR^, NCAM, and nAChR) with co-migration confirmed for p75^NTR^ (Hislop et al., 2014). However, despite transport being rapid and effective, neuronal transduction was comparatively inefficient, suggesting that post-trafficking processes are suboptimal in neurons (Hislop et al., 2014). Upon arrival at the cell body, LV must undergo a process known as uncoating, in which several viral proteins (e.g., Gag structural proteins) are removed to permit reverse transcription of the viral RNA (Matreyek and Engelman, 2013). The resulting double-stranded DNA then complexes with virus proteins for entry into the nucleus *via* the nuclear pore complex, before integration into the DNA of the host neuron. Improving understanding of these processes in motor and sensory neurons will be key to optimizing the effectiveness of intramuscular virus delivery.

## Influence of Pathology

**Figure 2:**
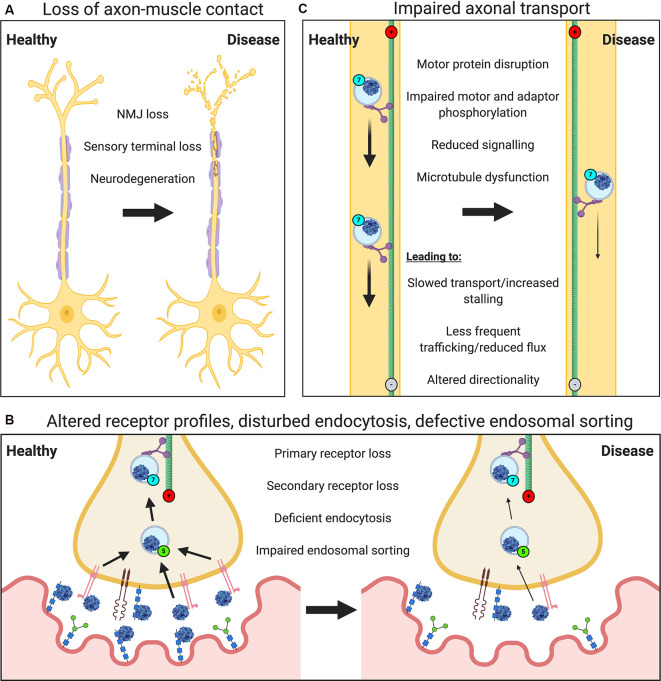
Neuropathological events impair the viral transduction of peripheral neurons. Several general and virus-specific pathological events caused by neurological disease diminish the effectiveness of gene therapy delivery to the nervous system *via* muscle. **(A)** Loss of motor and sensory nerve endings due to neurodegeneration will restrict nerve-muscle connections and the frequency of virus-nerve interaction. **(B)** Alterations in the expression or availability of certain primary or secondary receptors will affect virus attraction and binding. Deficits in endocytosis, as seen in spinal muscular atrophy (SMA), or impaired endosomal sorting, as identified in amyotrophic lateral sclerosis (ALS) and some forms of Charcot-Marie Tooth disease (CMT), could reduce virus uptake into peripheral nerve terminals. Defects in Golgi processing and nuclear import may also decrease viral transduction (not depicted). **(C)** A variety of impairments affecting axonal transport machinery (e.g., microtubule dysfunction) are known to cause defects in cargo trafficking (e.g., slowed transport or reduced quantity/flux), which will limit viral delivery.

Neuropathology will impact most, if not all, major steps in the journey of viruses from the nerve terminal to the nucleus (Figure 2). Neurodegeneration of peripheral nerves results in the loss of axon terminals within muscles (Figure 2A). Motor neuron retraction from the NMJ, i.e., denervation, is an early feature of motor neuron diseases [e.g., ALS, SMA and Charcot-Marie-Tooth disease (CMT; Goulet et al., 2013; Moloney et al., 2014; Sleigh et al., 2014; Spaulding et al., 2016)], and will limit neuron-virus interactions within muscles. Sensory degeneration observed in conditions like CMT (Sleigh et al., 2017), will have a similar restrictive effect. Nonetheless, motor neurons branch frequently within muscles resulting in multiple contacts across the entire muscle; thus, if one or several NMJs become denervated, there is likely to be a window of time in which at least some neuromuscular contacts of a pathological neuron remain viable. In ALS mice, for instance, rather than all neuromuscular contacts of a single motor neuron denervating simultaneously, healthy synapses close to degenerating NMJs are more likely to denervate than those located further away, suggestive of localized pathological transfer (Martineau et al., 2018). It is therefore conceivable that functional synapses may facilitate virus uptake and nuclear delivery to preserve the integrity of NMJs that remain. Moreover, once delivered, viral vectors encoding secretable proteins (e.g., neurotrophins) can influence central networks through both autocrine and paracrine mechanisms (Baumgartner and Shine, 1997; Benkhelifa-Ziyyat et al., 2013). NMJs resident in different muscles, and even within a single muscle, can show large differences in both pre- and post-synaptic structures (Mech et al., 2020) as well as levels of key synaptic proteins (Allodi et al., 2016), thus virus binding and uptake are likely to differ across motor nerve terminals.

Significantly, intramuscular injections of gene therapy viruses can result in efficient and extensive transgene expression within the neonatal and adult mouse spinal cord, brainstem, and sensory ganglia, likely *via* the cerebrospinal fluid (Benkhelifa-Ziyyat et al., 2013; Chen et al., 2020). This finding is particularly important, as it suggests that injecting one muscle can result in viral transduction of an array of central neurons (Chen et al., 2020), meaning that not all muscles require injection for potential widespread motor and sensory neuron transduction; although injecting more muscles can cause greater therapeutic benefit (Benkhelifa-Ziyyat et al., 2013). Furthermore, muscle transduction can be used to promote synaptogenesis and/or reinnervation after neuromuscular pathology (Darabid et al., 2014). In this regard, collateral sprouting and dynamic remodeling of the NMJ, as is observed in ALS mice (Martineau et al., 2018), may also be therapeutically targeted.

In addition to the loss of peripheral nerve endings in muscle, deficiencies in endocytosis (e.g., in SMA; Dimitriadi et al., 2016), endolysosomal sorting (observed in many conditions; Neefjes and van der Kant, 2014), Golgi processing (e.g., in ALS; van Dis et al., 2014), and nuclear import (e.g., in ALS; Dormann and Haass, 2011) would all likely reduce the efficiency of viral transgene expression (Figure 2B). As would pathology-associated restrictions in axonal transport (Figure 2C), which have been reported in many neurodevelopmental and neurodegenerative conditions (Sleigh et al., 2019), such as the signaling endosome transport deficits observed in ALS mice (Bilsland et al., 2010; Sleigh et al., 2020a). Nevertheless, Rab7-positive endosomes containing AAV have been shown in primary cortical neurons *in vitro* to increase retrograde transport speeds compared to non-AAV containing Rab7 organelles (Castle et al., 2014b), which could perhaps counteract transport dysfunction.

Only a few studies are have investigated the impact of disease on virus transduction after intramuscular delivery. Despite downregulation during development, CAR expression is upregulated in regenerating adult skeletal muscle in response to disease (Nalbantoglu et al., 1999; Shaw et al., 2004; Sinnreich et al., 2005), which will likely positively impact AdV uptake. Increased levels of sialic acid, a known AAV9 inhibitor, in the CNS of a mouse model of lysosomal storage disorder have been shown to severely limit the effectiveness of AAV9-mediated gene therapy (Chen et al., 2012). Nonetheless, the opposite may be true for particular AdV and other AAV serotypes, which use sialic acid as a primary attachment factor. Involved in pro-apoptotic signaling during development, but downregulated in the mature nervous system, the p75^NTR^ receptor is also re-expressed in neurons after disease or trauma (Dechant and Barde, 2002), possibly impacting LV efficacy. For example, p75^NTR^ expression is increased in SOD1^G93A^ mice motor neurons and human ALS tissue (Lowry et al., 2001), and plays a key role in organizing and maintaining NMJ connectivity (Pérez et al., 2019). Moreover, NCAM expression is a major regulator of synaptic remodeling in pre-synaptic NMJ terminals (Chipman et al., 2014) and levels are dysregulated in ALS (Jensen et al., 2016), which could also affect LV binding. Also, the background of the experimental animal can influence the transduction efficiency of some vectors and must be carefully considered (He et al., 2019). Overall, these studies warn against the assumption of similar virus binding and uptake profiles between healthy and disease states and indicate that further studies in disease models at symptomatic stages are required.

Despite these hurdles, intramuscular injections of gene therapies have proved successful at symptomatic stages in ALS mice (Tosolini and Sleigh, 2017), hence the above-discussed effects of pathology do not abolish virus transduction. Furthermore, symptomatic SMA patients treated with *onasemnogene abeparvovec* to augment SMN protein levels respond positively to treatment (Mendell et al., 2017), albeit with AAV administered intravenously. Nevertheless, while it remains unclear precisely how and to what extent specific diseases and associated pathologies will impact the transduction of peripheral neurons, the described viral vectors have the undisputed potential for the treatment of neuromuscular disorders when delivered to skeletal muscle.

## Optimizing Intramuscular Gene Therapy

One of the biggest challenges facing gene therapy is achieving sufficient delivery to target cells/tissues to combat disease. This is particularly difficult for peripheral nerve disorders in which pathological cells are located deep within the spinal cord and behind the BBB and BSCB. Several investigator-independent factors such as nervous system maturity (Foust et al., 2009; Tosolini and Morris, 2016a) and pathology influence viral transduction and transgene expression, but these cannot be modified in a clinical setting. However, varied investigator-driven factors also impact the effectiveness and should be carefully considered when designing gene therapy for intramuscular administration. Differences in tropism, infectivity, and transport between viruses and their serotypes will impact the success of this delivery method; for example, in a side-by-side comparison, muscle injection of rAAV2-retro was shown to have superior capacity to transduce peripheral neurons compared to AAV serotypes 1, 2, and 5–9 (Chen et al., 2020). Similarly, superior LV pseudotypes based on hybrid glycoproteins have also been identified (Hirano et al., 2013; Eleftheriadou et al., 2016). Moreover, vector purity and concentration will impact transduction levels (Hollis Ii et al., 2008; Klein et al., 2008), as will the efficiency and specificity of the promoter (von Jonquieres et al., 2013; Borel et al., 2016), the choice of which can also reduce off-target expression, and hence further enhance therapeutic potential (Parr-Brownlie et al., 2015).

Several different methods have been pioneered that can enhance peripheral neuron transduction upon intramuscular virus administration. As may be expected, these techniques focus on enhancing virus uptake rather than other processes essential to transduction. For instance, a complementary viral strategy can be used to boost the expression of the virus receptor(s) at peripheral nerve terminals that can then be therapeutically targeted with a different virus, as has been demonstrated with AAV-mediated CAR expression for increased AdV binding and uptake (Larochelle et al., 2010; Li et al., 2018b). Receptor expression may also be selectively increased by genetic overexpression (Nalbantoglu et al., 2001) or administration of drugs that enhance transcription, albeit non-specifically (e.g., histone deacetylase inhibitors; Larochelle et al., 2010). Similarly, genetic screens are beginning to identify a variety of viral restriction factors (i.e., proteins that constrain uptake and transduction), which could also be genetically or chemically manipulated, perhaps in a tissue-specific fashion, to aid uptake (Mano et al., 2015; Madigan et al., 2019). Alternatively, approaches are being developed in which recombinant viral receptor proteins are conjugated to biomaterials and pre-loaded with gene therapy viruses before injection. Indeed, intramuscular administration of recombinant cysteine-tagged AAVR chemically linked to polyester microspheres and pre-incubated with AAV resulted in local and prolonged gene delivery with reduced spread compared to AAV alone (Kim et al., 2019). However, it remains to be seen whether this system can be adapted to increase uptake into peripheral nerve terminals, which would require the release of AAV from the receptor microspheres. Similarly, viral capsids can be chemically modified with a variety of different substances that may aid peripheral nerve binding (e.g., conjugation with neuron-specific homing peptides; Terashima et al., 2009), or antibodies against key neuronal receptor proteins (e.g., p75^NTR^ and CAR; Hedley et al., 2006; Eleftheriadou et al., 2014). Furthermore, motor neuron transduction efficiency upon intramuscular administration of AdV was shown to be enhanced by pre-treatment with flaccid paralysis-causing botulinum toxin type A (BoNT/A; Millecamps et al., 2002). Likely mediated by enhanced motor terminal sprouting, this enhancement was even greater in the SOD1^G93A^ ALS mouse (Millecamps et al., 2001, 2002).

Unfortunately, many of these strategies are not currently a clinical possibility, for obvious reasons. Nonetheless, their implementation in the laboratory to deliver genes within the therapeutic range, along with the development of novel and improved tools to assess virus transduction and treatment efficacy (Han et al., 2019; Chen et al., 2020; Sleigh et al., 2020b; Surana et al., 2020; Ueda et al., 2020), will undoubtedly lead to improved understanding of disease mechanisms and assessment of potential gene therapy strategies.

## Conclusion

Gene therapy injected into the skeletal muscle for delivery to neurons holds therapeutic promise for peripheral nerve disorders. Motor and sensory nerve terminals located within muscles can act as therapeutic conduits not only for the innervating neurons (Figure 1) but also neighboring nerve and glial cells *via* paracrine mechanisms. Moreover, some viruses can escape from the initially transduced neurons, resulting in widespread gene delivery throughout the spinal cord, brain stem, and sensory ganglia. Importantly, this indicates that not all muscles need to be injected to obtain broad cellular dosing. Unfortunately, neuropathology is likely to hinder the effectiveness of intramuscular gene therapy delivery (Figure 2); but innovative pre-clinical methods are being developed that will enhance peripheral neuron transduction *via* this method. Also, the intramuscular administration could be combined with, for example, intrathecal delivery to further enhance CNS uptake. However, due to the immune response, repeated successful dosing is unlikely, and hence such treatments need to be given within a short time frame to circumvent this impediment. Nevertheless, by factoring in a detailed understanding of the dynamics of viruses and host cell receptors, especially in the context of peripheral nerve biology and neuromuscular pathology, perhaps this minimally invasive delivery method can contribute to successful gene therapy in the future.

## Author Contributions

AT and JS wrote the manuscript and have approved the submission of this work.

## Conflict of Interest

The authors declare that the research was conducted in the absence of any commercial or financial relationships that could be construed as a potential conflict of interest.
